# How Do Parents With Bipolar Disorder Perceive the Risk to the Next Generation? Results From a Qualitative Study

**DOI:** 10.1111/bdi.70139

**Published:** 2026-06-24

**Authors:** Michelle Laigaard, Christina Wagner, Stine Pedersen, Julie Ravneberg Stokholm, Maj Vinberg

**Affiliations:** ^1^ The Early Multimodular Prevention and Intervention Research Institution (EMPIRI), Psychiatric Centre, North Zealand Copenhagen University Hospital–North Zealand Hospital Hillerød Denmark; ^2^ Copenhagen Affective Disorders Research Centre (CADIC) Psychiatric Center Copenhagen Frederiksberg Denmark; ^3^ Department of Clinical Medicine, Faculty of Health and Medical Sciences University of Copenhagen Copenhagen Denmark

**Keywords:** bipolar disorder, children, heritability, high‐risk family, qualitative research

## Abstract

**Objectives:**

There are several high‐risk studies of children of parents with bipolar disorder (BD); however, the impact of being an “At‐Risk” parent has seldom been studied. The aim is to explore how parents with BD perceive their potential heredity and how they feel toward their children being invited to take part in high‐risk family studies.

**Methods:**

Qualitative semi‐structured face‐to‐face interviews were conducted with 14 participants. All interviews were audio recorded, transcribed verbatim, and analyzed inductively using qualitative content analysis by researchers with different professional backgrounds. Data collection continued until no new themes emerged, suggesting data saturation.

**Results:**

Five main themes were identified: (1) parenting outweighs genetics, (2) guilt—the emotional burden of parenting with bipolar disorder, (3) self‐stigmatization, (4) challenges in communication within the family, and (5) reflections on participation in research. Parents often took on the role of a Bipolar‐Detective, monitoring their child's mental health and took precautions hoping to prevent them from developing BD. Subtle differences were noted between mothers' and fathers' reflections.

**Conclusion:**

This was a qualitative study of selected families with BD parents; the findings should therefore be interpreted with caution. The participants were concerned about their children's well‐being due to BD, emphasizing their own parenting challenges over genetic risk. Future studies should address parental fears alongside treating BD and offer guidance on communicating with their children. While most viewed high‐risk family study participation positively, some worried about potential pathologizing of their children. These aspects are critical and noteworthy when engaging with children from high‐risk families.

## 1 Introduction

Bipolar disorder (BD) ranks among the most prevalent mental illnesses worldwide, characterized by an early onset and heightened risk of recurrence [1]. BD is associated with diminished overall functioning and quality of life, as well as increased morbidity and mortality rates [2]. Individuals with BD, and their close relatives, often face societal stigma, including within work, family, and social circles, compounded by self‐stigmatization wherein negative beliefs and stereotypes about one's illness are internalized [3, 4].

Familial aggregation is evident in affective disorders, with twin studies showing a substantial genetic component [5]. Children of parents with BD are at significantly elevated risk for developing various mental disorders over their lifetime [6, 7]. Thus, studies of high‐risk families have gained prominence [8, 9]. The onset of BD commonly occurs between the ages of 12 and 24 years [10]. Extensive research focuses on children and teens whose parents have affective disorders, aiming to identify those at risk of serious mental health issues [11, 12]. Early comprehensive intervention strategies involving the entire family are recommended [13, 14]. Parents with BD often have concerns about their children's mental well‐being [15]. However, most high‐risk children will not develop psychopathology before puberty. Parenting teenagers is challenging; having BD intensifies these concerns. There is a lack of understanding and insight into the parents' experiences, worries, coping mechanisms and transparency regarding the impact of their illness on their offspring [15]. Supporting and helping the parents concerning these worries might also help their children [13].

Psychoeducation is a well‐established component of treatment for BD, shown to enhance illness insight, improve medication adherence, and reduce relapse rates [16]. However, most psychoeducational approaches have targeted individuals with BD or the family unit, with limited attention to the specific experiences, needs, and concerns of BD. Research addressing how psychoeducation can support these parents in managing parenting‐related worries, discussing mental illness with their children, and coping with perceived intergenerational risk remains scarce.

It is thus of clinical interest to investigate (1) how parents with BD perceive the intergenerational risk of mental illness regarding their adolescent children, and (2) how they perceive their adolescents' potential involvement in research interviews and at‐risk assessments.

### 1.1 Aims of the Study

Our study aims to explore how parents with BD perceive the intergenerational risk of mental illness and how they feel about their children being invited to take part in high‐risk family studies.

## 2 Material and Methods

### 2.1 Design

A qualitative study based on semi‐structured interviews and analyzed using qualitative content analysis as described in the guidelines by Elo and Kyngäs [17].

### 2.2 Participants

Fourteen patients (thirteen with bipolar disorder and one with recurrent depression) receiving treatment at the Affective Disorder Clinic, North Zealand, Denmark, were included. Eligible parents had an ICD‐10 [18] diagnosis of bipolar disorder (type I or II) or recurrent depressive disorder who had ≥ 1 child aged 13–25 years and spoke Danish. Participants were identified by clinicians and invited during routine visits, with purposive sampling to ensure variation in sex and family situation. The patients with recurrent depression were included due to its clinical and familial relevance. The sample's demographics reflected the clinic's typical caseload.

### 2.3 Interviews

A semi‐structured interview guide (Supplementary 1), developed by the research group, was used. Throughout the interviews, the interviewer, M.L. or C.W., sought clarification of meanings, encouraged elaboration on various viewpoints, and redirected participants if their discussion veered too far from the central topics [19]. Additionally, the participants completed a short battery of questionnaires after the interview assessing mood and parental stress (see Supplementary 2).

All interviews were audio recorded and took place in a research room at Mental Health Centre Hillerød (from February to June 2024). The interviews lasted between 27 and 107 min and the information was anonymised.

### 2.4 Data Analysis

The analysis group consisted of an MD (M.L.), an MD, Post Doc (C.W.), MD (J.R.S.), and a Master of Arts in Philosophy of Education (S.P.). Both M.L. and C.W. are experienced in clinical psychiatry. J.R.S. contributed to the development of the study protocol and provided feedback on data analyses and the final manuscript. Furthermore, S.P. has lived experience within the field of analysis due to herself being a patient with BD as well as a mother to an adolescent. Thus, the researchers had different experiences and preunderstandings on the topic. None of the researchers had any preexisting knowledge of the participants. The interviews were transcribed verbatim (M.L.). Data analysis was structured using the qualitative research software Nvivo [20].

**FIGURE 1:**
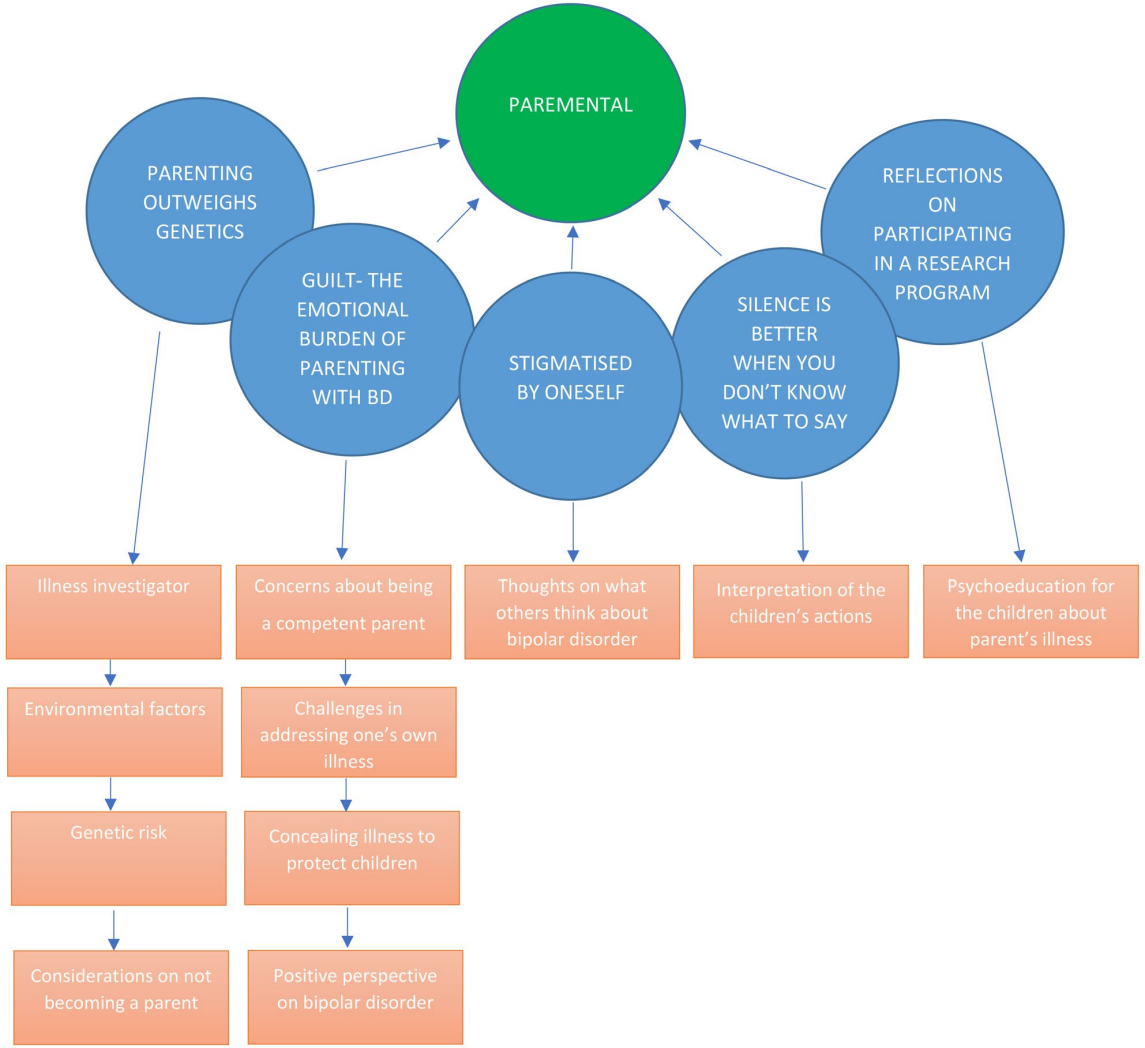
The five themes and their corresponding subthemes based on the qualitative interviews.

The analysis consisted of five steps: Step 1, “Open Coding”: Each researcher read the interviews independently marking all meaning units. A meaning unit was defined as a segment or word that could illuminate the research questions. All meaning units were assigned a preliminary code defined by the researcher describing the content. Step 2, M.L., C.W., and S.P. discussed preliminary codes until agreement on the meaning of the content. Step 3, Preliminary codes were merged into new codes until no further meaningful merging was possible. Step 4, Five themes were predefined according to the major topics of questions in the interview guide (Figure 1). The content and meaning of all codes were organized into themes and subthemes. Step 5, M.L. wrote the description of the themes in accordance with the discussion, and the written description was revised by the other researchers until agreement. M.L., C.W., and S.P. discussed for a shared understanding while ensuring a minimum of interpretation and focus on the phrases used by the participants [21]. Analysis was conducted continuously while conducting interviews.

## 3 Findings

### 3.1 Participant Characteristics

We included 14 participants, eight women and six men. Table 1 shows the characteristics of the participants. The Major Depression Inventory score generally indicated a normal level of mood (Mean = 18.6; standard deviation [SD] = 12.33) [22]. The Altman Mania Self‐Rating Scale indicated no sign of significant symptoms of mania (Mean = 2.42; SD = 2.38) [23]. Further quantitative measures are shown in Supplementary 2.

**TABLE 1:** Participant characteristics.

Patient	Sex	Age	Employment status	Relationship status	Children (age)
1	Female	41	Sick leave	Married	2 (11, 13)
2	Male	57	Sick leave	Married	2 (19, 22)
3	Female	49	Sick leave	Divorced	3 (15, 22, 24)
4	Female	48	Sick leave	Married	3 (13, 17, 20)
5	Female	46	Employed	Divorced	2 (12, 19)
6	Female	37	Job seeker	Divorced	2 (10, 16)
7	Female	53	Sick leave	Divorced	3 (16, 25, 27)
8	Female	45	Job seeker	Divorced	3 (7, 11, 18)
9	Male	46	Sick leave	Married	2 (15, 18, 26)
10	Female	44	Employed	Married	4 (7, 10, 12, 18)
11	Male	61	Employed	Divorced	1 (24)
12	Male	59	Sick leave	Married	2 (16, 19)
13	Male	58	Employed	Divorced	2 (13, 16)
14	Male	43	Employed	Married	2 (9, 13)

### 3.2 Sex Differences

During the analysis of the interviews, we observed a tendency in the responses of men and women, with men demonstrating less reflection on the topics they were asked to elaborate upon. They were less proficient in articulating their emotions and appeared less engaged in understanding their children's feelings, even though, when questioned, it seemed the topic had a significant impact on them. It is important to note, however, that given the small number of participants, these findings should not be interpreted as representative or generalizable, but rather as indicative of possible tendencies warranting further investigation.

### 3.3 Findings

Five themes were defined, each representing an overall question raised during the interview. Subthemes within each theme are presented (Figure 1).

- “Parenting outweighs genetics”—vigilant observation and responsibility for children's well‐being

Well actively seeking out symptoms, both those I'm already familiar with and any new ones I might have learned about. I'm trying not to criticize myself if I notice something concerning. I keep quiet about it, but I carefully observe and pay close attention. (Participant 3)

### 3.4 Illness Investigator and Observer Concerning the Children's Mental Well‐Being

Participants vigilantly observed their children for signs of BD. They described how they perceived symptoms in many of their children's insignificant actions. Due to their fear of their children developing the illness, they interpreted minor behaviors as indicative of potential illness, seeing signs in everything: “But of course, I keep an eye on things because I know there's a hereditary component. So, I'm attentive if she were to experience anything or become depressed or completely lose it, but she's also a teenager, you know.” (Participant 3).

When discussing this theme, many participants expressed and expanded upon their feelings of guilt and shame regarding the potential inheritance of their illness, which led them to search for symptoms and keep an extra eye on their children. Many participants felt they had inadvertently passed on a sensitive mental condition to their children, which heightened their sense of responsibility to intervene promptly upon noticing any signs of BD.

### 3.5 Environmental Factors

Participants had a hard time dealing with the fact that the way they lived their life with BD could in some way affect their offspring: “Well, it's kind of like this: I know it's an illness, and back then, I didn't know it would happen, but I can worry that on the days when there's a lapse in care and I'm feeling unwell, it might pass on to them genetically, but also emotionally harm them. Especially because so much is happening at their age right now, and many things can affect them. So, there's a lot of guilt and shame associated with this.” (Participant 5). When asked by the interviewer, participants acknowledged the fragility of their psyches stemming from their own childhood experiences.

Many described a spectrum of diagnoses prevalent in their familial lineage, such as autism, ADHD, depression, and BD, leading them to speculate that their offspring may inherit similar conditions. They described this as a confluence of factors, attributing the potential inheritance of a fragile psyche to their family history, coupled with the impact of having a parent who has experienced severe illness due to BD, which they fear could predispose their offspring to develop BD as well: “I've felt that my upbringing has influenced me significantly, and my children have also experienced a difficult upbringing because of my illness. So, in that sense, it could contribute to something happening?” (Participant 1).

### 3.6 Genetic Risk

The participant's thoughts about genetic risk were a part of one of the two main questions raised in the aim of the study. Participants generally attributed their mental health more to family environment than genetics, showing greater concern about passing BD to their children than their own heredity. Most had not thought much about genetic risk, focusing instead on environmental factors in transmission. However, some did acknowledge genetic concerns for their children, with one even considering not having children due to this risk: “I actually talked to my doctor about it and said that I didn't really want to have kids. So, the doctor asks, ‘Oh, why not?’ And I say, “Those genes, I really don't feel obligated to pass them on” (Participant 2).

### 3.7 Considerations on Not Becoming a Parent

Many participants mentioned that they had considered not having children due to their BD, particularly when discussing the heritability of the illness. They expressed awareness of the potential stress it could cause them and the impact on their well‐being. Additionally, they were concerned that their disorder might prevent them from being effective and good parents: “I said when my wife became pregnant, I wanted her to have an abortion. And I said that I saw a bleak future for myself if we had children, knowing that it would be hard for me”. (Participant 13).

II Guilt—the emotional burden of parenting with bipolar disorder.

You know you're depressed but don't seek help, you know you're manic but can't control it. That lack of control or the feeling of lack of control over oneself, it's unbearable. Because you sit with so much guilt afterwards. So even if I've only slept 3 hours during the day, I still feel guilty. (Participant 6)

### 3.8 Concerns About Being a Competent Parent

Due to their illness, participants frequently experienced feelings of inadequacy or deficiency in their parental roles. There was a profound sense of guilt and shame surrounding this matter, as it evoked substantial concern regarding the potential impact of their BD on their children when they could not consistently uphold the role of a competent parent: “When I'm really down. Then I can just do what needs to be done, they get food, and I can help a bit with homework. But when it's really bad, even that stops completely, and I can't muster anything. But over time, I've become more relaxed about it because they know I'm ill, but I'm worried about the lack of care.” (Participant 5).

### 3.9 Challenges in Addressing One's Own Illness

Participants expressed considerable difficulty in addressing their illness with their children worrying about how to talk about this appropriately. Moreover, they grappled with the dual roles of being both a parent and a patient: “I find it difficult to be a mother and a patient at the same time. On one hand, I want to comfort etc., but on the other hand, it's also about me.” (Participant 1). A component of the worries regarding discussing their illness with their children also stemmed from the participants' fear or distress at the prospect of their children expressing concern for them. Several articulated that it would be emotionally challenging for them to confront any indication that their children might be impacted by their BD. Consequently, they avoided broaching the subject altogether to shield their children from potential distress.

### 3.10 Concealing Illness to Protect Children

Participants collectively emphasized their practice of “hiding” their illness as a means of protecting their children. This tendency was driven, in part, by their dislike of having their children worry over them during episodes of illness. Such actions encompassed implementing modest adjustments in their daily routines or deliberately withdrawing from scenarios where the illness might become apparent: “I always make sure not to be lying on the sofa when they come home. That's when I empty the dishwasher or hang up laundry, or whatever it may be.” (Participant 4). The participants believed this quality was crucial in their daily lives, as it gave them strength as a parent, thereby removing concerns from their family members. Additionally, most of the parents expressed that reducing some of the symptoms in their daily lives could potentially decrease their children's fixation on symptoms of BD inherited from their parents. Notably, single parents among the participants encountered more significant challenges in establishing a stable and “normal” daily routine. Some expressed that this challenge was more pronounced for them, as they lacked a co‐parent who could provide support or step in when they were having a difficult day or required a break.

### 3.11 Positive Perspective on Bipolar Disorder

Several participants discussed the positive aspects of being a BD parent. They noted that tasks could be accomplished more easily during hypomanic periods, and they perceived themselves as more playful and cheerful around their children during these times: “I think it makes me a more fun parent, also because when you become a little hypomanic, you also become a bit childish.” (Participant 8). Many participants saw benefits in hypomania but were aware of the depressive episodes that often followed. They reported increased guilt and shame during these times, noting that depression made them more present by limiting distractions: “She has also experienced me during my depressive episodes, but to a lesser extent because that's when I'm actually more present” (Participant 11).

III Stigmatized by oneself.

But that's how I feel, that's how I am, that people look askance at me and they… think, she can't be a proper mother when she has depression, so she can't take proper care. Or take care of herself, so she can't take care of her children either. And nobody has ever said anything. It's all going on in my head. (Participant 6)

This theme was particularly prominent among the participants because many were very concerned about how they were perceived by others who knew about BD. “Well, it's because if someone has knowledge about what used to be called manic depression in the old days, they get compared a lot, or people think I'm about to do something crazy any minute. It's not nice.” (Participant 4). The participants were highly attentive to how others would perceive their disorder. Many expressed concerns that if people relied solely on information from the internet or media, they might form a misconstrued image of them as bipolar. In this regard, participants overwhelmingly desired a professional to educate their children about BD. This would ensure that nothing would be explained that could frighten them or lead to a negative interpretation of the disorder. These concerns regarding how their children perceived BD were so prevalent, which meant that several tried to compensate or hide their illness from their children: “I'm hiding the fact that I'm being hospitalized.” (Participant 7). Participants thought about how others perceived them as parents with mental illness: “And sometimes I feel like I must convince others that I'm a good parent. For example, I might go overboard packing their lunches to show others that I'm a good parent. Well, people shouldn't think I'm a stressed wreck who's just in and out of the hospital, so I overcompensate by trying to appear normal.” (Participant 5). When participants were asked whether they had experienced stigmatizing situations, most answered that they hadn't. On the contrary, they mentioned that people were kind to them and accepted them as regular individuals. As such, the concerns about stigmatization were primarily internalized by the participants, rather than reflecting actual experiences in society. The apprehension of being labeled as a bipolar patient was described as a self‐generated fear by the participants, rather than a tangible reality: “No, because I couldn't feel it in them. It was ideas in my head.” (Participant 2).

IV Silence is better when you don't know what to say.

'X has also asked why I take all those pills, and I've just explained it, you know. But I'm afraid they might think I'm dying or something. (Participant 4).

This is a central finding and an unmet need in supporting parents with bipolar disorders, starting a conversation with their child about what it is to have a parent with mood disorder.

The participants formed their conclusions about the children's concerns based primarily on their behavior in everyday life rather than actual conversations: “…it's more the way they accept that there's sandwiches for dinner. In the past, they might have complained about dinner, but they can sense it when I'm not feeling well and give me space.” (Participant 6). Many participants mentioned they thought their children had accepted their illness because it had become a part of their everyday life, and therefore had adapted to a normalization of the abnormal. “Yes, but they haven't known anything different. So, the entire perception of normalcy shifts. It just becomes part of the whole. It becomes a normal picture of what that family entails, and you just adapt to it” (Participant 12).

Few participants discussed their disorder with their children, often avoiding the topic due to guilt or shame over perceived parental inadequacy. Many also believed children struggled to share negative feelings. Despite limited conversations, parents sensed their children's concern through behaviors like avoiding certain topics or turning to the co‐parent for support. One participant, for example mentioned that her child would avoid discussions with the parent about specific topics or rely more on the co‐parent, all to protect the ill parent, which was interpreted as the child was concerned: “She's probably afraid of upsetting me, I think, so she'd rather skip some things than make her mom sad” (Participant 5). When directly questioned whether the participants felt their children were concerned, most responded that they were uncertain or unaware, because they hadn't asked their children directly.

V Reflections on participation in a research project.

Well, I think it's absolutely wonderful, and that's also why I agreed to come here and talk with you, because I also believe that there's a lack of material and knowledge, and those are some of the things we want to know, like have I infected anyone? Will my children also have this illness? (Participant 3)

This quote illuminates the study's second aim, in which participants were directly asked about their reflections and feelings regarding their children being asked to participate in high‐risk family studies. The central theme that emerged in the discussion was “Psychoeducation for the children, about parents' illness.” This was in greater demand among all the participants than focusing solely on the risk or hereditary factors. All participants meant that if their children had more knowledge about their illness, it could help them to understand their parents better and alleviate some of the anxiety and worry that the parents have: “Just like there's psychoeducation for adults about their illness, it would be nice if there were also some for the children so they could understand what's happening with their parents” (Participant 6).

Also, it was essential for the participants that the message was transmitted by a professional due to a general concern that the story of being bipolar would be passed on in the wrong way and cause worry among their children.

Overall, the participants didn't feel able to adequately explain about their illness and to talk about this with their children themselves. In general, most of the participants had a positive attitude toward including their children in a high‐risk study. One participant was directly opposed to this: “Well, I don't think it would be nice for my children if they were suddenly told that my illness might be hereditary. I don't think I want that to be examined” (Participant 7). Several also mentioned conflicting attitudes toward research projects, as they were worried about pathologizing their children. The participants feared triggering the disorder in their children if too much emphasis and focus were placed on symptoms and heredity, or if the children themselves became more aware of the illness and therefore developed worry toward their parents or themselves: “Yes, why delve into things that aren't an issue, why create a problem if it's not there? Let's deal with it when it arises” (Participant 5).

## 4 Discussion

In the present qualitative study, including 14 parents with BD, we found five themes representing the participants' attitudes toward the hereditary nature of their illness and their children being invited to participate in family studies for high‐risk families. The parents express fear regarding the possibility of passing on their disorder to their children. This concern was amplified by challenges of parenting with BD, which can significantly impact the well‐being and stability of their offspring. Along this vein, another study [24] suggests that the tumultuous and unstable everyday environment surrounding a parent with BD could predict emotional difficulties in the child. A notable finding was parents' varying degrees of symptom monitoring. A prominent focus was directed toward behavior, which they conscientiously endeavored to discern and demonstrated acute attentiveness toward in their children. Finally, some indications of sex differences were observed, with males appearing less reflective and more reluctant to express their concerns about their offspring.

Although the opening question of all interviews concerned their thoughts about their genetic risk, all participants started by explaining how they had become an “illness investigator,” rather than talking about their genetic risk. Instead, they expressed apprehension regarding the various non‐genetic factors that could potentially predispose their children to develop an affective disorder. Numerous participants described how they had developed a constant sense of apprehension, an “anxiety‐gene,” regarding various behaviors exhibited by their children. These observations are in line with another study regarding parents with BD [15]. This might indicate that despite their concerns for their children, their awareness of the impact of their moods could paradoxically empower them to perceive a degree of control over their offspring's well‐being [15]. This study found that the parents articulated their inability to control their actions and worries concerning their children [15]. The parents further highlighted their capacity to compensate by adapting their parenting skills and adjusting the family environment to meet the needs of their family better [15].

In general, the most prominent theme in the qualitative interviews was *Guilt—the emotional burden of parenting with bipolar disorder*. Many participants expressed the challenge of balancing their roles as both a parent and a patient simultaneously, feeling inadequate and deficient in their ability to care for their children. Consequently, they harbored concerns that this lack of adequate care might predispose their offspring to illness. This finding is recognized in other studies as well, which is noteworthy when advising bipolar parents in their parenting journey [25].

An intriguing observation in our study was the minimal communication between participants and their children regarding the parent's illness, as well as the perceptions and feelings of the children about having a parent with BD. Based on their observations, the participants expressed that they believed their children experienced worry. Yet, they found it challenging to directly inquire whether this concern was a reality for their offspring. Along this line, another study [26] revealed that children worry about ill parents but may hide these concerns to avoid adding to the parent's burden. Contrary to the initial assumption, the same study also observed that children would articulate their worry through verbal comments, support, and physical contact with the ill parent. They also note that children express a sense of inadequate access to information and guidance regarding their parent's illness, which can contribute to misunderstandings and frustrations within their families [26]. The participants in our study also mentioned this aspect and could recognize this in their household. The desire for more information is a shared need and aspiration among parents and children, aiming to acquire a common language to understand the parent's illness and enhance the family environment at home [27]. This aspect is also emphasized in another family study [13] that similarly advocates for increased access to information and the implementation of interventions within families with parental mental health issues. Their study revealed significant improvements in family functioning and child behavior following such interventions, underscoring their efficacy and importance. This tendency is also seen in other literature [24, 28].

Stigmatization emerged as a significant concern and many emotions among all participants, as they feared potential negative perceptions of their illness from family, strangers, other parents, or even their children. Indeed, additional literature also identifies a correlation between BD and stigmatization [29]. However, there is a difference from previous literature in our study, as the participants reported that they had not personally encountered stigmatization when questioned about it. They said that stigmatization was confined to their thoughts or perceptions. This finding suggests a potential need to support and educate bipolar patients through psychoeducation to reduce their internalized stigmatization and help their mental health [29].

Our participants represent both sexes, comprising eight women and six men. During the analysis of the interviews, a coherence was observed in how the male participants expressed themselves consistently throughout the interviews. They demonstrated less reflection on the topics they were asked to elaborate on and generally resisted expressing their feelings toward their offspring. Most of the male participants also struggled with accepting their BD and acknowledging its potential limitations on their way of life. The female participants were more accepting and aware of how to manage their disorder. Although this tendency is based on a small number of participants, making it difficult to draw definitive conclusions. However, the finding is also supported by additional literature [30], which underscores the urgent need for support strategies for male parents in coping with BD and helping them with parenting their children.

Our study asked the participants about their willingness to participate in a high‐risk study targeting their young offspring (aged 13–25 years). Here, most of the participants expressed a desire for their children to have access to additional information or psychoeducation concerning their parents' illness. They believed that such knowledge could facilitate their children's ability to cope with their illness more effectively [31]. The participants also preferred that a professional convey the message and knowledge about their illness. This preference came from concerns about their ability to communicate about BD to their children. They feared they might inadvertently say something incorrect, leading to heightened fear or stigmatization among their offspring. This observation, distinct from findings in other literature [32], is notable because participants expressed that knowing their illness had been communicated respectfully and accurately would give them a sense of relief toward their children. This finding could also serve as a valuable component to incorporate into early interventions for families affected by BD.

### 4.1 Strengths and Limitations

The qualitative themes analysis was conducted by investigators with different experiences and preunderstanding. One researcher had their own lived experience. This ensured different perspectives and helped keep a descriptive focus as close to the participants' own words and phrasing as possible [19, 21]. We consider this a strength of the study. Our study also included quantitative questionnaires to give a more accurate picture of how the participants answer our interview questions according to their mood. Our participants were included from one specialized affective disorder clinic, limiting variation. Our study comprises a group of only 14 participants; we did however not observe significant variation in the participants' responses to the research question after the 14 interviews, which suggests the presence of data saturation. In addition, the relatively small sample size of 14 participants increases the risk of selection bias, as those who chose to participate may differ in important ways from those who did not. The near‐balanced sex distribution offers insight into perspectives of both mothers and fathers.

## 5 Conclusion

This study is the first to qualitatively explore how parents with bipolar disorder perceive risks to their children and family‐based research. Parents were more concerned about the effects of their own emotional instability and parenting challenges than about genetics. These findings point to key areas for psychoeducation: addressing parental concerns about parenting, building parental confidence, and guiding communication about BD. Parents expressed willingness to participate in family research, hoping it would foster open mental health discussions within the families. Integrating psychoeducation into such research may boost engagement and benefit families, helping refine programs to meet both informational and emotional needs.

## Funding

This work was supported by Lundbeck Foundation (R432‐2023‐94).

## Supporting information


**Supplementary 1.** Interview guide translated from Danish to English.

**Supplementary 2.** Quantitative findings (Mn; SD) from the six questionaries.

## Data Availability

The data that supports the findings of this study are available in the Supporting Information of this article.
