# Supplementary material for: How Do Parents With Bipolar Disorder Perceive the Risk to the Next Generation? Results From a Qualitative Study
Source: Bipolar Disord. 2026 Jun 24;28(5):e70139. doi: 10.1111/bdi.70139 (PMC13292732; doi:10.1111/bdi.70139)
Supplement: Supplementary file 1 — Supplementary 1. Interview guide translated from Danish to English. [file BDI-28-0-s001.docx]

*Supplementary 1*

Interview guide translated from Danish to English.

| **Section** | **Suggested Phrasing and Elaborative Questions** | **Expected time 1 time 15 min (max 90 min)** |
| --- | --- | --- |
| **Introduction (by the Interviewer)** | - The interviewer introduces themselves (again) by name and title. - Briefly explains the purpose of the interview and its framework, reminds the participant that the interview is being recorded, and assures confidentiality and anonymity. - Mentions that confidentiality may be waived in cases of immediate risk of self-harm or harm to others, where it is obligatory to inform relevant psychiatrists or authorities for safety. - Obtain informed consent. | 3 min |
| Basic Information about the Participant | - "To start, could you briefly tell me a bit about yourself, how old you are, and what you do on a daily basis?" - If the participant is on sick leave/unemployed, ask further about previous work experience and possibly education. - If the participant has a different ethnic/cultural background, inquire about this as well. | 5 min |
| Background and Diagnosis History | - "This interview focuses particularly on being a parent with a mental illness, either depression or bipolar disorder. Could you briefly describe your own illness?" - "Did you grow up in a family where there was a history of mental illness?"   Here, we are interested in describing factors such as severity, duration, functional level, hospitalizations, and family history. Explore the family history in terms of severity (mild mental disorders, severe affective or psychotic disorders), suicide, and substance abuse. | 5 min |
| Information about Children | - "Please tell us a bit about your children, what they do, and how they are doing." - Supplement with additional questions about the number of children, their gender, and ages if the participant does not mention it themselves. Inquire whether they live at home or together. - If the participant indicates that the children show signs of illness, explore this further. - "How is your daily interaction with your children?" | 5 min |
| Heredity and Transmission of the Illness | - "What thoughts have you had about the heredity of your illness?" - "How do you deal with the heredity of your illness in daily life?"   (Preventive measures, awareness of children's symptoms)   - "Beyond heredity, what thoughts have you had about whether your illness affects your children?" | 10 |
| Reflections on Parenting Role and Stigmatization | - "What thoughts have you had about being a parent with depression or bipolar disorder?" - An elaborative question, if the participant does not mention it themselves: "Were these things you thought about before becoming a parent?" - "What impact do you think your illness has on you as a parent?" - "Have you thought about whether your illness affects your children?" - "Have you considered what your partner or co-parent thinks about your illness concerning your children or you as a parent?" - "Is this something you have discussed?" If so, elaborate on what the partner/co-parent has said. - "Have you considered what others, in general, think about your illness in relation to your children or you as a parent?" | 15 |
| Children's Reflections | - "Families vary in how and how much they talk about mental illness. How do you handle this in your family?" - "What thoughts have you had about what your children think about your illness?" - "What thoughts have you had about what your children think about their own risk of developing the same illness? - "Is this something you have discussed with your children?" If so, elaborate on what the children have said. | 10 |
| Reflections on Children's Participation in Research on Risk of Developing Illness | - "What do you think about researchers wanting to study the risk of illness in children of parents with mental illness?" - "What do you think it would mean for your family if your children participated in a research project on the risk of developing mental illness?" - If the participant does not mention any disadvantages: "Can you imagine any disadvantages of conducting research on children of parents with mental illness?" | 15 |
| Conclusion | - "We are about to wrap up for today, and I think we have discussed some very interesting topics. Finally, is there anything you thought of that you would like to share that we have not discussed today?"   It is important to only open up new topics if they are relevant to the study. If the question is more for the interviewer or another subject, close the interview by saying, "I would like to follow up on that afterwards, but for now, let's conclude the interview and say thank you."   - Thank the participant for their participation. After thanking, turn off the recorder to signal the end of the interview. | 5 min |
