# Supplementary material for: How Do Parents With Bipolar Disorder Perceive the Risk to the Next Generation? Results From a Qualitative Study
Source: Bipolar Disord. 2026 Jun 24;28(5):e70139. doi: 10.1111/bdi.70139 (PMC13292732; doi:10.1111/bdi.70139)
Supplement: Supplementary file 2 — Supplementary 2. Quantitative findings (Mn; SD) from the six questionaries. [file BDI-28-0-s002.docx]

Supplementary 2

Quantitative findings (Mean; Standard Deviation (SD)) from the six questionaries.

| Scale | Mean ± SD |
| --- | --- |
| MDI | 18.6 ± 12.33 |
| Altman Mania Self- rating Scale | 2.42 ± 2.38 |
| Parental Stress Scale | 61.57 ± 4.37 |
| CTQ | 61.12 ± 5.19 |
| FAD | 153 ± 9.5 |
| Loneliness Scale | 54.2 ± 3.08 |

The participants were invited to complete six questionnaires. The Major Depression Inventory score generally indicated a normal level of mood (Mean= 18.6; SD=12.33)^1^. The Altman Mania Self-Rating Scale indicated no sign of significant symptoms of mania (Mean= 2.42; SD=2.38)^2^. Parental Stress Scale indicated that the participants experienced elevated levels of stress (Mean= 61.57; SD=4.37)^3^. The participants showed a history of childhood trauma (Mean= 61.12; SD= 5.19) when completing the Childhood Trauma Questionnaire^4^ . The Family Assessment Device results revealed that the participants exhibited severely pathological family functioning (Mean= 153; SD= 9.5)^5^. Finally, the participants experienced a moderately high degree of loneliness, according to Loneliness Scale (Mean= 54.2; SD= 3.08)^6^.

1.Bech P, Rasmussen NA, Olsen LR, Noerholm V, Abildgaard W. The sensitivity and specificity of the Major Depression Inventory, using the Present State Examination as the index of diagnostic validity. J Affect Disord. 2001;66(2-3):159-164. doi:10.1016/S0165-0327(00)00309-8

2. Altman EG, Hedeker D, Peterson JL, Davis JM. The Altman Self-Rating Mania Scale. Biol Psychiatry. 1997;42(10):948-955. doi:10.1016/S0006-3223(96)00548-3

3. Berry JO, Jones WH. The Parental Stress Scale: Initial Psychometric Evidence. http://dx.doi.org/101177/0265407595123009. 1995;12(3):463-472. doi:10.1177/0265407595123009

4.. Bernstein DP, Stein JA, Newcomb MD, et al. Development and validation of a brief screening version of the Childhood Trauma Questionnaire. Child Abuse Negl. 2003;27(2):169-190. doi:10.1016/S0145-2134(02)00541-0

5. Epstein NB, Baldwin LM, Bishop DS. THE McMASTER FAMILY ASSESSMENT DEVICE. J Marital Fam Ther. 1983;9(2):171-180. doi:10.1111/J.1752-0606.1983.TB01497.X

6. Hughes ME, Waite LJ, Hawkley LC, Cacioppo JT. A Short Scale for Measuring Loneliness in Large Surveys: Results From Two Population-Based Studies. Res Aging. 2004;26(6):655. doi:10.1177/0164027504268574
